# The Antagonist pGlu-βGlu-Pro-NH_2_ Binds to an Allosteric Site of the Thyrotropin-Releasing Hormone Receptor

**DOI:** 10.3390/molecules26175397

**Published:** 2021-09-05

**Authors:** Daniel L. De La Cruz, Laszlo Prokai, Katalin Prokai-Tatrai

**Affiliations:** Department of Pharmacology and Neuroscience, University of North Texas Health Science Center, Fort Worth, TX 76107, USA; DanielDeLaCruz@my.unthsc.edu (D.L.D.L.C.); Laszlo.Prokai@unthsc.edu (L.P.)

**Keywords:** allosteric binding, TRH antagonist, TRH receptor, homology model, hTRH-R, docking, extracellular domain, G protein-coupled receptor, surface-recognition binding

## Abstract

After we identified pGlu-βGlu-Pro-NH_2_ as the first functional antagonist of the cholinergic central actions of the thyrotropin-releasing hormone (TRH, pGlu-His-Pro-NH_2_), we became interested in finding the receptor-associated mechanism responsible for this antagonism. By utilizing a human TRH receptor (hTRH-R) homology model, we first refined the active binding site within the transmembrane bundle of this receptor to enhance TRH’s binding affinity. However, this binding site did not accommodate the TRH antagonist. This directed us to consider a potential allosteric binding site in the extracellular domain (ECD). Searches for ECD binding pockets prompted the remodeling of the extracellular loops and the *N*-terminus. We found that different trajectories of ECDs produced novel binding cavities that were then systematically probed with TRH, as well as its antagonist. This led us to establish not only a surface-recognition binding site for TRH, but also an allosteric site that exhibited a selective and high-affinity binding for pGlu-βGlu-Pro-NH_2_. The allosteric binding of this TRH antagonist is more robust than TRH’s binding to its own active site. The findings reported here may shed light on the mechanisms and the multimodal roles by which the ECD of a TRH receptor is involved in agonist and/or antagonist actions.

## 1. Introduction

**Figure 1:**
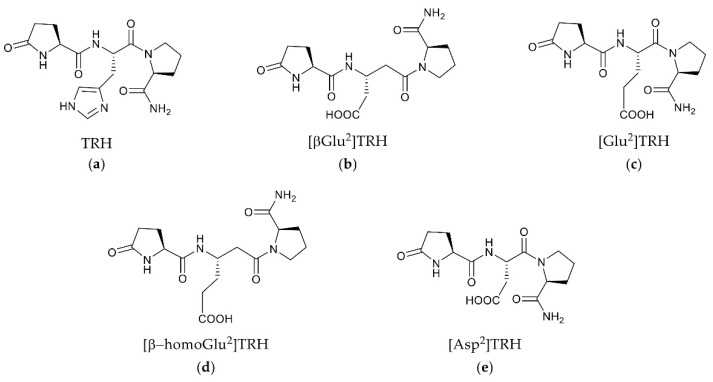
Chemical structures of TRH (**a**) and related pGlu-Xxx-Pro-NH_2_ peptides (**b**–**e**) in which Xxx is a central acidic amino acid residue (βGlu, Glu, β-homoGlu or Asp), while TRH possesses a basic central residue (Xxx = His).

Two forms of G protein-coupled receptors (GPCRs) have been identified for thyrotropin-releasing hormone (TRH, pGlu-His-Pro-NH_2_, Figure 1a) in the rodent brain: TRH-R1 and TRH-R2 [1]. These receptors are unevenly distributed throughout the body of murine species, with the highest expressions found, for example, in the brain’s limbic structures, the neuroendocrine brain regions and the frontal cortex [1,2]. Only TRH-R1 appears to be involved in the suprahypothalamic effects of TRH and TRH-like peptides [1,2,3,4]. In addition, the human brain has been found to express only a single TRH receptor similar to the rodent TRH-R1 [1,5]. The human TRH receptor (hTRH-R) is over 90% homologous to the TRH-R1 of murine species and is classified as a member of the seven-transmembrane domain (TMD) rhodopsin/adrenergic receptor subfamily of Class A GPCRs [5,6,7]. During our recent search for selective functional antagonists of TRH’s central actions, we identified pGlu-βGlu-Pro-NH_2_ ([βGlu^2^]TRH, Figure 1b) as a novel peptide that completely reversed TRH’s cholinergic effects in the rodent brain through neuropharmacological and neurochemical paradigms [8]. The potent antagonism exerted by 1b has yet to be explained in the context of its interaction with a TRH receptor. 

Although an experimentally determined structure of any TRH receptor remains to be seen, highly conserved Class A GPCR features have aided the development of a homology model for the hTRH-R based on the neuropeptide Y1 receptor [9]. In this model, the seven transmembrane regions are linked with three extracellular loops (ECLs) and three intracellular loops. The extracellular domain (ECD), including the ECLs and the *N*-terminus, has important roles in many GPCR functions. For instance, in bovine rhodopsin, the first GPCR characterized by X-ray crystallography [10], ECL-2 is capable of projecting into the transmembrane domain (TMD) to facilitate active site binding. In particular, this loop is also the largest and most versatile among the three ECLs with various functional roles in ligand binding. It also contains a cysteine residue (Cys) that is conserved across the GPCR superfamily to aid the structural integrity of the receptor by forming a disulfide bond with another Cys located elsewhere in the ECD [11]. Additionally, the seven α-helices of GPCRs contained within the cell membrane are arranged in a tertiary structure that resembles a barrel, with its cavity serving as a ligand-binding domain often covered by ECL-2 [10,12]. Many GPCRs also contain active binding sites within the ECD [11,12,13]. Although the hTRH-R active binding site is located within the transmembrane bundle (TMB) where α-helices form a binding pocket [14], TRH receptor-binding studies have demonstrated that interactions with the ECD are likely and may be similar to other GPCRs binding small biogenic amine ligands [14,15]. Overall, GPCRs have various conformations that range from inhibitory to fully activated states, where the distinct orientation of ECLs may be critical to receptor activity [13].

Nevertheless, ECLs are often inadequately resolved in crystal structures [11,12,13]. Therefore, computational techniques to create multiple receptor models with unique and energetically favorable ECL trajectories have become paramount in understanding how the ECD may influence ligand binding [12,16]. Presently, there is no consensus regarding ligand interactions within the ECD beyond experimental receptor-binding data from mutagenesis studies. The results of these investigations propose a surface interaction between TRH’s C-terminal pGlu residue and Tyr-181 of ECL-2 as integral for receptor activation, perhaps by mediating the formation of a TMB channel into the active binding site [14,15].

To understand the action of the newly identified TRH antagonist 1b (Figure 1) [8], the present study was devoted to the evaluation of the ECD of the hTRH-R and examination of its role in binding TRH versus 1b and related peptides with sequence pGlu-Xxx-Pro-NH_2_, where Xxx denotes Glu (Figure 1c), β-homoGlu (Figure 1d), or Asp (Figure 1e). In 1c–e, the acidic central residue is isomeric or homologous to βGlu in 1b, and these tripeptides were also included in our previous study that led to the identification of 1b as the first selective functional antagonist of TRH’s cholinergic actions in the rodent brain [8]. Reevaluation of a previously reported hTRH-R homology model [9] allowed us to identify a binding pocket at the receptor’s surface that serves as the actual ligand-recognition site for TRH to enter a TMB channel leading to receptor activation. While this binding site did not accommodate 1b, we identified an allosteric binding site that exhibits a selective and high-affinity binding for this TRH antagonist. This allosteric binding is more robust than our projected TRH binding to the active binding site within the TMB.

## 2. Results and Discussion

### 2.1. Refining the Active Binding Site of the hTRH-R 

When evaluating a recent homology model of TRH’s cognate receptor [9], the peptide arbitrarily docked within the hTRH-R binding pocket resulted in a free energy change (ΔG) of −7.0 kcal/mol and a binding affinity that was estimated in the mM range [17,18]. These initial findings are in agreement with a recently published study using a different hTRH-R homology model based on the turkey β-1 adrenergic receptor [19]. Both homology models produced TRH poses with only a partial fit into the proposed active binding site, which lies deeper within the TMB [20]. Additionally, these docking studies did not fully support the critical involvement of Tyr-106, Asp-110, Tyr-282, and Arg-306, as these residues have been proposed to be essential in TRH binding to its receptor [14,20].

**Figure 2:**
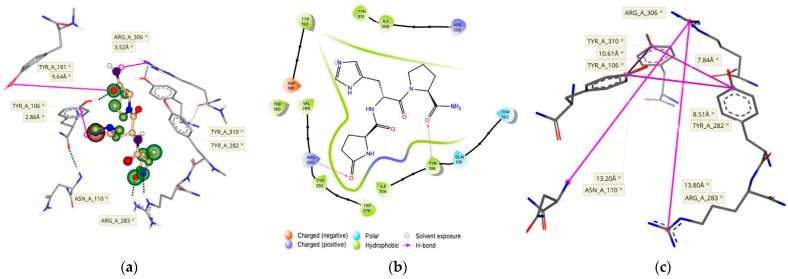
(**a**) TRH docked within the TMB of its receptor with an estimated binding affinity in the nM range. Hydrogen bonding is represented as green dashed lines, whereas the size and color of the coronas surrounding each ligand atom represent the degree of contribution of that atom to the binding score (green indicates favorable, red denotes unfavorable). (**b**) This binding is also outlined in a 2-D pharmacophore model. (**c**) The equidistant position of the Tyr residues on each of the three α-helices, as well as the vertical boundaries of the binding pocket are outlined with measurements made between key residues.

Therefore, we heuristically changed the spatial orientations of these residues at the active binding site of the receptor. Once we established a pocket with an adequate size to accommodate TRH below the protein surface at the TMB, we generated a TRH pose (Figure 2a) showing ligand interactions within 3–4 Å from the proposed key residues [14,20]. Additional residues proposed to be involved in the active binding site, Arg-283 and Tyr-310 [14,15,20], can also be seen in our model. The active binding site of the hTRH-R contains several polar amino acid residues to facilitate the strong binding of TRH. Furthermore, the peptide’s backbone interacts with the side chains of Ile-109, Trp-279 and Ile-309 (illustrated by large green coronas in Figure 2a), while directly hydrogen-bonding with Tyr-106 and Arg-283. Interactions of TRH within the mainly hydrophobic binding pocket are outlined in the 2D-pharmacophore representation (Figure 2b). In addition, we have found that the side chain orientation of TRH’s central residue is within 3 Å from Tyr-106, indicating that an aromatic interaction is also possible [21]. 

Interestingly, the three helices that form the hTRH-R active site each contain Tyr (Tyr-106, Tyr282, and Tyr-310) that encircles the binding pocket (Figure 2c). It has been suggested that TRH’s central His may interact with any of these residues located on each side of the binding pockets [14,15,20]. Therefore, TRH may engage in multiple, transient interactions with these aromatic residues as it moves within the pocket until it is locked into an optimal position [21]. In such a scenario, the C-terminal Pro-NH_2_ may better align with Arg-306 as it has been previously hypothesized [20], since the binding site is roughly 14 Å (in vertical dimension of binding pocket) from Arg-306 to Arg-283 and measures 8–10 Å between each of the Tyr residues (Figure 2c). Furthermore, Asn-110 has been proposed to interact with the *N*-terminal pGlu [9,14]. Our hTRH-R model also demonstrates that Asn-110 is in the same distance from Arg-306 as Arg-283, although not in a favorable position (Figure 2a) to interact with the pGlu’s carbonyl as speculated [14,20]. Altogether, as a result of our active binding-site pocket refinement shown in Figure 2, TRH’s binding affinity increased to the nM range with a concomitant decrease in ΔG (–8.1 kcal/mol). This is in sharp contrast to what we found upon creating an hTRH-R model based on the previously published homology model [9] that, again, exhibited an estimated binding affinity for TRH within the mM range. 

### 2.2. ECD Modeling of the hTRH-R

When the active binding site of TRH within the TMB was tested by utilizing a template-based docking method [18] for 1b as our peptide of interest, docking was not achieved. This prompted us to consider 1b’s effect a consequence of allosteric binding to the receptor. In searching for this allosteric binding site, the ECD of our refined hTRH-R model (Figure 2) was scrutinized to better understand its role in receptor activation. Some GPCRs (e.g., Class C) are distinguished by a large extracellular *N*-terminus that facilitates ligand-binding interactions in concert with ECLs and the TMD [11,22]. The ability of GPCRs to utilize *N*-termini and ECLs to facilitate ligand interactions makes GPCR-targeting a complex process. In this regard, it has been proposed that small ligands such as TRH may interact not only within the TMD of GPCRs but also have multiple binding contacts at the receptor’s surface leading into the active binding site. This is supported by mutagenesis and receptor-binding studies [9,14,15,20,23]. Speculation of two distinct TRH binding sites for the TRH receptor [14,20] prompted us to investigate a stepwise binding process that utilizes a binding pocket at the protein’s surface (i.e., a ligand recognition binding site) via interactions with the ECD, subsequently aiding the formation of a tunnel into the TMB. These interactions may produce a prerequisite conformation (i.e., receptor “pre-activation”) that facilitates a stepwise binding process and dissociation cascade for TRH to move it along a channel into the active binding site. 

**Figure 3:**
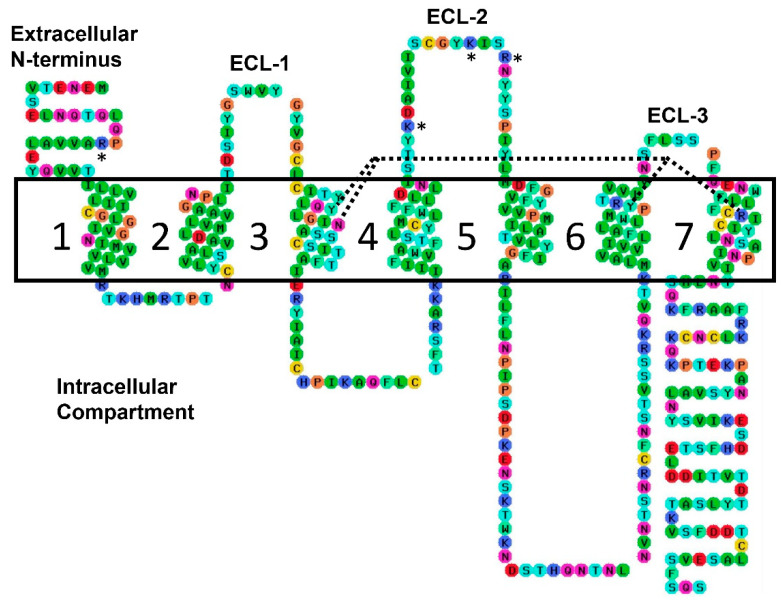
A two-dimensional outline of the hTRH-R peptide sequence pointing out the four direct binding contacts with Tyr-106, Asn-110, Tyr-282 and Arg-306 marked by dashed lines. The ECD’s positively charged residues are identified with asterisks. The TM helices are numbered (1–7), and the three ECLs are labeled. Color coding: red—Charged (negative), blue—Charged (positive), green—Hydrophobic, aqua—Aromatic, cyan—Hydrogen bond donor/acceptor, orange—Special cases, and yellow—Cys. The ECD’s positively charged residues are identified with asterisks.

The initially proposed hTRH-R homology model [9] visualizes ECL-2 as a hairpin loop with an upward or vertical trajectory relative to the TMD, which is believed to permit TRH’s access to the TMB active binding site. However, this orientation of ECL-2 does not support Tyr-181 on ECL-2 as critical to TRH recognition and high-affinity binding to the hTRH-R [14,15]. In fact, the position of Tyr-181 was initially modeled near the tip of the hairpin loop where it is completely solvent-exposed [9,14,15,24]. On the other hand, a previously reported hTRH-R homology model [19] possesses an ECL-2 that projects into the active binding site. This may be a result of utilizing the turkey β-1 adrenergic receptor as a template that contains an ECL-2 that is longer than that of the hTRH receptor. This homology model also neglected the disulfide bond that has been experimentally validated between Cys-98 of ECL-1 and Cys-179 of ECL-2 [14,19,25]. As such, this model also permits an increased mobility of ECL-2, thereby inaccurately proposing that ECL-2 can directly interact with the active binding site. In contrast, our model that successfully docked TRH within the TMB active binding site, involving Tyr-106, Asn-110, Tyr-282 and Arg-306 (Figure 2a,b and Figure 3), did not reveal any involvement of ECL-2 via Tyr-181, while still maintaining the disulfide bond between Cys-179 and Cys-98 in ECL-1. We propose that ECL-2 is situated directly above the defined active binding site (Figure 2a) and controls the receptor surface recognition of TRH. 

The multimodality and high flexibility of the GPCR ECD prompted us to utilize a modeling program that specifically focuses on protein loops [26]. Succeeding models depict the conformation of ECL-2 as aligning either vertically or horizontally across the TMD, essentially acting as a gating mechanism to a TMB channel that leads into the active binding site pocket of the hTRH-R. ECL-2 gating is a common trait for GPCRs and has been validated with GPCR’s crystal structures where the configuration of the ECD is dependent on ligand-binding modalities (e.g., agonist versus antagonist) [11]. However, the “open” state (i.e., vertical orientation of ECL-2) may not necessarily promote access to the TMB active binding site; rather this conformation may be the result of the receptor’s ligand-bound activation state as seen in the crystal structure of human protease-activated receptor 1, also a Class A GPCR [22]. 

**Figure 4:**
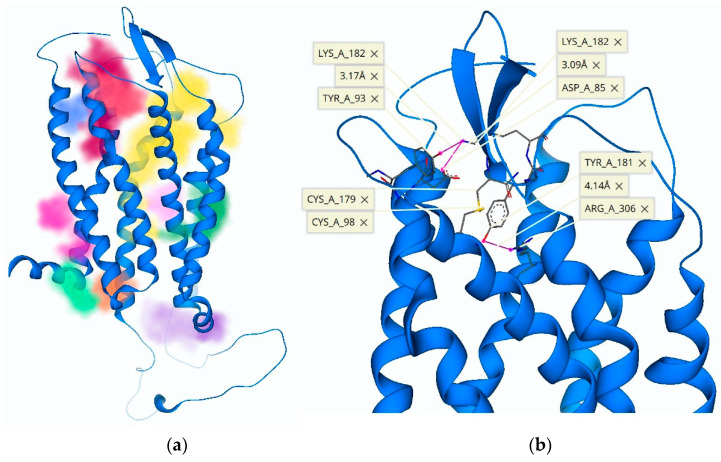
(**a**) The movement of ECLs creates unique binding pockets, highlighted in various colors. The binding pathway for TRH is shown in yellow and leads from the ECD down to the TMB active binding site. (**b**) ECL-2 is capable of interacting with multiple ECDs such as the *N*-terminus or other ECLs. Shown here is an ion–ion interaction between Lys-182 of ECL-2 and Asp-85 of ECL-1 that causes Tyr-181 to project downward towards the opening of the TMB channel.

During the creation of our hTRH-R homology models via ModLoop, the preservation of the disulfide bond between Cys-178 of ECL-2 and Cys-98 of ECL-1 consistently facilitated a loop trajectory that aided the ionic interaction of Asp-173 of ECL-2 with Arg-17 on the *N*-terminus, leading to an energetically favorable conformation. In our investigation, we also found that the nearest distance of ECL-2’s Tyr-181 to the active binding site of the hTRH-R was between 5–8 Å above Arg-310. Additionally, we found that ECL-2 orientations produce transient binding sites dependent on the configuration of the unique loop trajectories (Figure 4a). It has been proposed that polar and electrically charged residues of ECL-2, specifically residues Tyr-181 and Lys-182, form complementary electrostatic interactions with TRH [14]. This interplay with ECL-2 may attract TRH towards a surface recognition site that is formed by an ionic interaction between Lys-172 of ECL-2 and Asp-85 of ECL-1, as seen in Figure 4b. Altogether, extensive ECL modeling and docking experiments revealed key interactions within the ECD that not only facilitate the formation of the surface recognition site between ECL-1 and ECL-2, but also an inclination of Tyr-181 to mediate the formation of a TMB tunnel that leads from the protein’s surface into the active binding site (Figure 4b).

### 2.3. Surface-Recognition Binding Site of the hTRH-R

During ECD modeling, as described above, we created a library of energetically favorable conformations having various ECL and *N*-terminal trajectories. Accordingly, modifications of these extracellular components produced models with distinct binding pockets that depend on their configurations. It has been suggested that TRH interacts with the ECLs of the hTRH-R to expose a binding site on the protein’s surface, mediated by ECL-2 [9,14,20]. This TRH recognition produces a receptor conformation that allows TRH to progress into the active binding cavity along a TMB channel that is formed when ECL-2 is horizontally positioned across the TMD [12,14,16]. 

**Figure 5:**
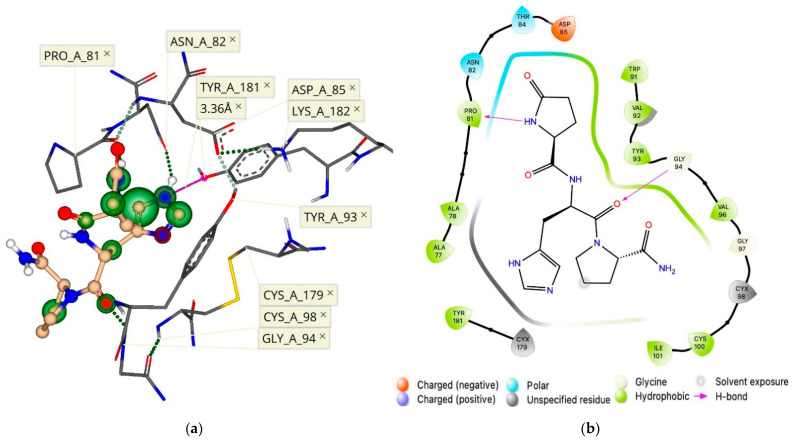
(**a**) Three-dimensional and (**b**) two-dimensional pharmacophore models of TRH docked to the proposed recognition binding site at the hTRH-R surface.

When we analyzed the newly created ECD binding pocket variations, we indeed identified a mechanism by which TRH is recognized at the protein’s surface via a pocket formed through an interaction between ECL-2 and ECL-1 (Figure 5). This surface binding-site affinity for TRH was estimated to be in the nM range. Here, Tyr-181 is positioned at the entrance of a channel that begins at the receptor’s surface and leads deep down into the TMB, where TRH is known to activate its receptor [14,15,20]. We argue that fluctuations of ECL-2 correspond with ligand interactions, occurring first between the polar and electrically charged residues of ECL-2 (Figure 3) in the open state to guide TRH into the protein’s surface-recognition binding site. Upon interaction with TRH, ECL-2 takes on a closed position, thereby promoting ligand recognition via a binding pocket produced when Asp-85 and Tyr-93 of ECL-1 interact with Lys-182 of ECL-2 (Figure 4b). Subsequently, Tyr-181 is then positioned at the top of the TMD active binding pocket pointing downward (Figure 4b). Oscillation among the ECLs may enable TRH’s movement along a channel into the TMB, where ligand binding may produce a conformation that relaxes ECL-2 back into an open state. Accordingly, interactions with other constituents of the ECD, such as the *N*-terminus, become inconsequential. Altogether, we report here that the hTRH-R surface recognition and activation via TRH results from the crucial roles of the ECD that may also contribute to the interpretation of agonist versus antagonist actions of TRH-related peptides.

### 2.4. Allosteric Binding Site of the hTRH-R

As mentioned previously, when the active binding site of TRH within the TMB (Figure 2) was tested for 1b utilizing template-based docking [18], no docked poses could be generated. This prompted us to consider 1b’s effect [8] as a result of allosteric binding to the receptor. Independently of the binding mechanism, a competitive inhibitor, an inverse agonist or an antagonist may bind to different sites, but the overall effect must prevent a TM helix orientation that exposes the intracellular G-protein binding site [11,12,13]. In this regard, if a substantial interaction occurs within the ECD prior to TRH recognition and is reinforced by the binding of an allosteric modulator, such as 1b, the development of TRH’s initial surface-recognition binding site may then be prevented.

In exploring the fundamental mechanism observed in accordance with our previous experimental data [8], transient binding pockets produced via remodeling of our hTRH-R homology model’s ECD were probed with TRH-related pGlu-Xxx-Pro-NH_2_ peptides shown in Figure 1b–e. These peptides contain an acidic side chain that increases the potential of these tripeptides to form strong ion-ion interactions with the receptor’s basic amino acid residues. In our search for an allosteric binding site, the hTRH-R sequence was evaluated for positively charged residues on the ECD that could form ionic interactions with 1b–e. We found only four such residues that are exposed above the protein’s surface, as highlighted in Figure 3. Interestingly, three of these residues are located on ECL-2 (Lys-172, Lys-182 and Arg-185), with the fourth situated on the *N*-terminus (Arg-17). 

**Figure 6:**
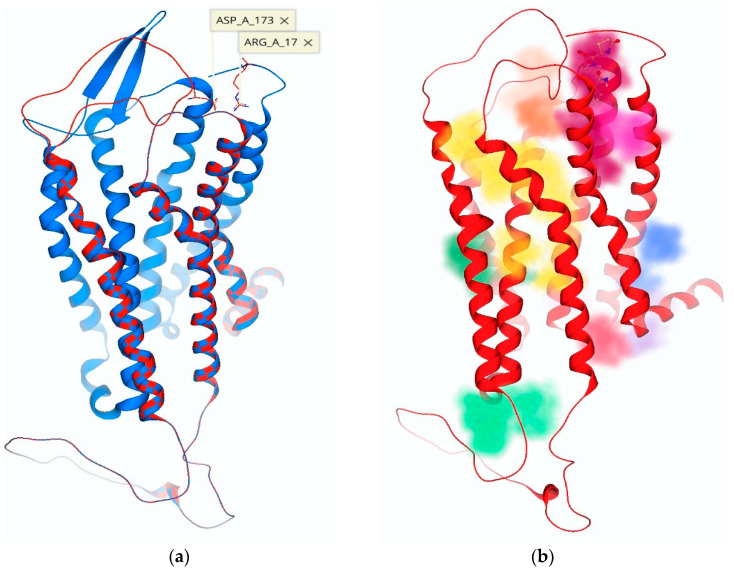
(**a**) The three-dimensional structure of the TRH receptor with an overlay of ECL-2 in the “open” (blue) versus the “closed” (red) position, following ECL-2 remodeling. (**b**) Pocket detection software highlights possible binding sites with various colors: the antagonist 1b (Figure 1) is shown docked in its allosteric binding site (magenta), and the TMB channel leading to the active binding site is highlighted in yellow.

Consequently, we evaluated TRH receptor conformations for the creation of transient binding pockets within the ECD that utilized these residues of interest. During this process, we recognized an ionic interaction between ECL-2 (Asp-173) and the *N*-terminus (Arg-17). Upon further analysis of these conformations, the development of binding sites was monitored via pocket detection algorithms based on a heuristic model and Gaussian differences within a three-dimensional (3-D) grid to determine the likelihood of a binding cavity (Figure 6) [27,28]. The pockets we created could not be realized with the initial model of ECL-2 in the vertical position [9]. Our proposed allosteric binding site was the product of a receptor conformation that produced a surface binding pocket of adequate size and with complementary electrostatic properties. Thusly, the accommodation of TRH analogues (Figure 1b–e) was facilitated by three electrically charged residues (Arg-17, Lys-172 and Asp-173) in close proximity (Figure 6a).

**Figure 7:**
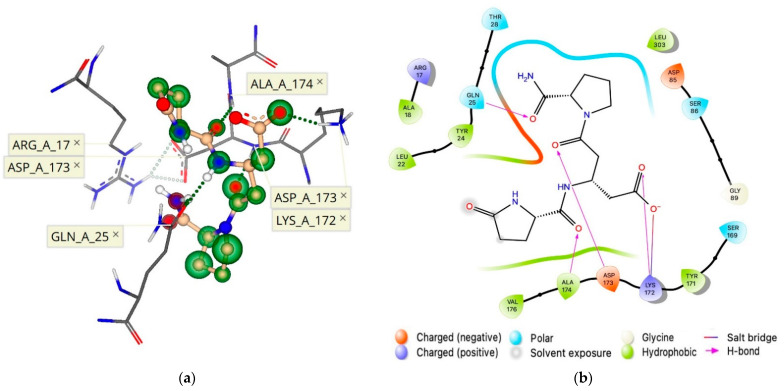
(**a**) A three-dimensional and (**b**) a two-dimensional pharmacophore model of 1b (Figure 1) docked to the proposed allosteric binding site of the hTRH-R homology model with an estimated binding affinity in the nM range (Table 1).

Upon probing novel ECL binding sites, molecular docking experiments initially utilized 1b ([βGlu^2^]TRH; Figure 1), the most inhibitory of the TRH analogues shown in Figure 1b–e [8], to determine which unique receptor conformation formed a high affinity and selective allosteric binding pocket to bind 1b (Figure 6b). Indeed, this binding is facilitated by several pharmacophore points, as shown in Figure 7. We established that the allosterically bound ligand-receptor complex of 1b produced a binding affinity estimated in the lower nM range and with a calculated ΔG of –8.8 kcal/mol (Table 1). Accordingly, 1b had a greater binding affinity for its allosteric binding site relative to TRH’s affinity for the hTRH-R’s active binding site (ΔG of –8.1 kcal/mol). 

Subsequent analysis using 1b as a template within this allosteric binding site (Figure 7), produced a ranked affinity for 1c–e (Figure 1), shown in Table 1. TRH was excluded from docking, as “no pose could be generated” within the binding site within the ECD. These predictions are in agreement with our experimental data in terms of the cholinergic agonist versus antagonist effects in the rodent brain [8]. Specifically, 1c–e could only partially antagonize TRH’s cholinergic effect. This was shown by a neuropharmacological assessment addressing the peptide’s ability to reverse pentobarbital-induced sleeping time (called analeptic effect). Additionally, TRH’s ability to stimulate extracellular acetylcholine turnover in the rodent brain [29] was also modestly antagonized by 1c–e. On the other hand, 1b was capable of completely antagonizing TRH’s cholinergic effect in the rodent brain [8]. 

The orientation of the ECD constituents has been shown to influence GPCR activity [7,11,12,13], and our data revealed that an hTRH-R allosteric binding pocket is created when ECL-2 interacts with the extracellular *N*-terminus (Figure 6) and selectively binds 1b (Figure 7). When ligand 1b is bound to the receptor, induced-fit docking of TRH into the TMB active binding site resulted in a reduced binding affinity. Furthermore, this proposed receptor conformation of 1b bound to the allosteric binding site depicted Tyr-181 of ECL-2 pointing away from the TMD. Therefore, TRH ligand recognition cannot be facilitated by an interaction with Tyr-181 on ECL-2, according to this initial ECL-2 trajectory. An antagonist that strongly binds to this allosteric binding site within the ECD of the hTRH-R, such as 1b, effectually disrupts the suspected gating mechanism of ECL-2, an essential domain pivotal to a variety of GPCR functions, by locking the ECD in a conformation that prohibits TRH recognition at the receptor’s surface. Inhibition was further supplemented by the high affinity and selectivity of 1b compared to TRH.

In conclusion, we identified for the first time a novel allosteric binding site within the ECD of our proposed hTRH-R homology model (Figure 7), which allowed the interpretation of 1b’s antagonism of the cholinergic action by which TRH exhibits its plethora of CNS effects. We also refined the TMB active binding site of TRH (shown in Figure 2), which illustrated the peptide’s dynamic movement within the binding cavity. Furthermore, our investigation led to the recognition of a surface binding site for TRH (Figure 5) that facilitates the formation of a TMB channel allowing TRH’s access to the hTRH-R active binding site.

## 3. Materials and Methods

### 3.1. hTRH-R Homology Model

Similar to the previously described model [9], a 3-D homology model of the hTRH-R was created via SWISS-MODEL, an automated homology modeling server that can be freely accessed at http://swissmodel.expasy.org on 13 June 2021 [30].

#### 3.1.1. Template Identification 

The input sequence of the hTRH-R (UniProtKB/Swiss-Prot entry: P34981) is available from the Universal Protein Resource (http://www.uniprot.org accessed on 13 June 2021) [31]. This target sequence was used to identify homologous protein structures within the PDB database as templates and to obtain target–template alignments via searching the SWISS-MODEL Template Library, which utilizes both the Basic Local Alignment Search Tool (BLAST) [32] and the Hidden Markov model (HMM-HMM)-based lightning-fast iterative sequence search (HHblits) [33]. Over 600 templates were found to match the target sequence with a sequence similarity greater than 30% (standard sequence homology to produce an adequate homology model).

#### 3.1.2. Homology Model Building

The SWISS-MODEL homology model was built based on target template alignment, utilizing the ProMod3 modeling software [34]. In order to return a complete protein structure, this modeling engine, in concert with other software, generates coordinates and sidechain conformations for amino acids by a template scaffold alignment, models sequence regions without template coverage, resolves stereochemical irregularities via energy minimization of a molecular mechanics’ force field, and performs insertion-deletion modeling [34]. To develop our hTRH-R homology model, we chose the neuropeptide Y1 receptor (PDB code: 5ZBH) [35] as the template X-ray crystal structure [9]. The resulted homology model was downloaded as a PDB file from the SWISS-MODEL web server and visualized with the SeeSAR software suite (version 11.0.2; BioSolveIT GmbH, Sankt Augustin, Germany) [18]. Superimposition of our model with a precalculated hTRH-R homology model, via SeeSAR, was used to evaluate the alignment of the seven transmembrane α-helices. This precalculated hTRH-R model, retained within the SWISS-MODEL Repository (https://swissmodel.expasy.org/repository/uniprot/P34981 accessed on 13 June 2021) [36], employed the human kappa opioid receptor crystal structure (PDB code: 4DJH) as its template [37]. Only highly scrutinized homology models are imported into the SWISS-MODEL Repository, thus, the precise alignment of the transmembrane helices between this precalculated model and our own homology model, along with similar model quality assessment scores using SWISS-MODEL evaluation checks (GMQE and QMEAN), was a promising indication of our modeling approach [34,36]. Furthermore, both models also had 95% of dihedral angles in sterically acceptable regions via Ramachandran plot statistics.

### 3.2. ECD Modeling 

Recognizing the large flexibility that ECLs exhibit, ModLoop, a web-server based extension of Modeller computational chemistry software was utilized to produce various loop confirmations [26]. The loop trajectories were scored based on ModLoop’s molecular dynamics algorithms with the most energy-favorable structure returned as the output. The following amino acid residues were closely monitored in the initial screening of binding pockets to interact with the hTRH-R active binding site [14,19,20]: Tyr-106 and Asn-110 in TM-3, Tyr-282 and Arg-283 in TM-6, and Arg-306 and Tyr-310 in TM-7. Additionally, the key interactions monitored within the ECD included Arg-17 in *N*-terminus, Cys-98 in ECL-1, Lys-172, Cys-179, Tyr-181, Lys-182 and Arg-185 in ECL-2, Tyr-192 at protein surface, and Asn-289 in ECL-3. The amino acids selected for loop remodeling are shown in Figure 3; effectually, all residues contained in the ECD were subjected to remodeling in various combinations except for Cys-98 and Cys-179 in order to maintain their critical disulfide interaction.

### 3.3. Docking 

TRH docking into the active binding site of our developed hTRH-R homology model was initially performed using AutoDock Vina and AutoDock Tools software (San Diego, CA, USA) [17]. Following ECD remodeling, each of the numerous receptor conformations created contained unique binding pockets that were assessed with SeeSAR software to determine the ligand binding affinity of selected compounds (Figure 1). Docking results with the highest binding affinity were visualized via Schrödinger Suite 9.0.02 software package (New York, NY, USA) [38] to produce a two-dimensional pharmacophore of the docked complexes. The ΔG of the bound complex was evaluated with AutoDock Vina for comparison with the initial screening of the hTRH-R active binding site. Binding energies obtained by AutoDock Vina are represented as ΔG of the total bound complex compared to the unbound receptor [17]. Using our hTRH-R homology model, the AutoDock Vina docking grid (20 × 20 × 20 Å box) was defined as centroid to residues Tyr-106, Trp-279, Tyr-282 and Arg-306; additionally, rotatable ligands and a flexible-receptor docking protocol specifying these residues were also employed. Other docking parameters were set to default.

#### Ligand and Protein Setup, Docking Protocol, and Identification of Binding Sites 

The chemical structure of TRH and related peptides (Figure 1) was drawn within SeeSAR’s graphical user interface, where they were optimized through an automated process to assign the proper geometry, protonation state and tautomeric form in preparation for molecular docking. Similarly, as the hTRH-R homology models were imported into SeeSAR, downloaded via the ModLoop web server in PDB file format, they were optimized using an empirical scoring function to automatically generate a hydrogen-bonding network, as well as assign atom type and amino acid ionization states. To establish a docking protocol with SeeSAR software, the docked pose with the lowest ΔG of TRH bound to its receptor’s active binding site produced by AutoDock Vina was imported in PDB format. The ligand was removed, and prior to redocking the binding pocket was manually refined by rotating the sidechains of Tyr-106, Asn-110, Trp-279, Tyr-282 and Arg-306 to expand the active binding-site cavity. This binding site was defined based on a 6.5 Å shell around the position of the removed ligand. Molecular docking was performed with SeeSAR’s FlexX docking and HYDE scoring algorithms, which involved the fragmentation of the ligand in order to reconstruct it incrementally within the binding cavity, assigning free energy of binding scores (ΔG) to each of the ligand’s atoms depending on the emerging hydrogen bond and dehydration energies of the receptor-ligand complex [39,40]. Ultimately, SeeSAR software allowed the docking and visualization of ligand poses within the binding pockets of each unique hTRH-R conformation, and it assessed binding affinities in terms of concentration ranges of the Kd dissociation constant for the ligand binding [18]. The highest affinity docked pose of each ligand (with the appropriate protein-ligand interactions) was selected based on its SeeSAR binding prediction and was imported into AutoDock Vina for scoring [17] to allow a subsequent comparative evaluation of ΔG with our initial docking experiments.

Following the explicit ECD remodeling of our hTRH-R and side-chain manipulation at the active binding site, as described above, nearly 200 hTRH-R homology models were visualized in SeeSAR. These unique conformations were searched for the unoccupied and transient binding pockets produced as a result of the movement of ECLs and the *N*-terminus via ModLoop. Detection of binding sites was based on the DoGSite algorithm that analyzes a pocket’s geometric and physicochemical properties and estimates drug-like properties [28]. For each ligand of interest (Figure 1), a maximum of 500 poses was generated in SeeSAR and docked to every identifiable binding pocket within the TMB and ECD of the diverse receptor conformations developed from ECD modeling of our hTRH-R homology model. Favorable ligand poses with the greatest calculated binding affinities to our newly discovered binding sites, described herein, were precisely analyzed using the Schrödinger Suite 9.0.02 software package [38].

## Figures and Tables

**Table 1:** Binding affinities of TRH (Figure 1a) and TRH-like peptides (Figure 1b–e) within the proposed allosteric binding site of the hTRH-R homology model. K_d_ denotes dissociation constant.

Ligand	Binding Affinity (ΔG, kcal/mol)	K_d_
TRH (1a)	– ^1^	– ^1^
[βGlu^2^]TRH (1b)	−8.8 ± 2.5	nM
[Glu^2^]]TRH (1c)	−6.1 ± 2.5	nM–µM
[β-homoGlu^2^]TRH (1d)	−5.6 ± 2.5	μM–mM
[Asp^2^]TRH (1e)	−5.4 ± 2.5	µM–mM

^1^ N/A (no binding pose could be generated).

## Data Availability

All data sets are available upon reasonable request from the corresponding author.

## Sample Availability

Samples of the compounds are not available from the authors.
